# Role of vocal tract characteristics in individual discrimination by Japanese macaques (*Macaca fuscata*)

**DOI:** 10.1038/srep32042

**Published:** 2016-08-23

**Authors:** Takafumi Furuyama, Kohta I. Kobayasi, Hiroshi Riquimaroux

**Affiliations:** 1 Graduate School of Life and Medical Sciences, Doshisha University, Kyotanabe, Kyoto, Kyotanabe, Kyoto, 610-0321, Japan

## Abstract

The Japanese macaque (*Macaca fuscata*) exhibits a species-specific communication sound called the “coo call” to locate group members and maintain within-group contact. Monkeys have been demonstrated to be capable of discriminating between individuals based only on their voices, but there is still debate regarding how the fundamental frequencies (F0) and filter properties of the vocal tract characteristics (VTC) contribute to individual discrimination in nonhuman primates. This study was performed to investigate the acoustic keys used by Japanese macaques in individual discrimination. Two animals were trained with standard Go/NoGo operant conditioning to distinguish the coo calls of two unfamiliar monkeys. The subjects were required to continue depressing a lever until the stimulus changed from one monkey to the other. The test stimuli were synthesized by combining the F0s and VTC from each individual. Both subjects released the lever when the VTC changed, whereas they did not when the F0 changed. The reaction times to the test stimuli were not significantly different from that to the training stimuli that shared the same VTC. Our data suggest that vocal tract characteristics are important for the identification of individuals by Japanese macaques.

Many studies have suggested that primates, including humans, can identify individuals by listening to their vocalizations. The pygmy marmoset (*Cebuella pygmaea*) recognizes other group members as individuals^1^. Rendall and colleagues demonstrated that rhesus macaques (*Macaca mulatta*) can also distinguish the species-specific communication “coo calls” of kin from those of non-kin and distinguish among the coo calls of close kin using a habituation–dishabituation paradigm^2^. Adult squirrel monkey (*Saimiri sciureus*) mothers are able to distinguish the voices of their own infants from those of other juvenile individuals^3^. Several other species, including vervet monkeys (*Chlorocebus pygerythrus*)^4^, Japanese macaques (*Macaca fuscata*)^5^, and rhesus macaques^6^, also exhibit the ability to identify their infants based on voice alone. These studies indicate that the identification of individuals by their vocalizations is important for many primates.

Despite the behavioural significance, there are still debates regarding how non-human primates identify individuals from their vocalizations and about the neural mechanisms underlying individual vocal identification. Most monkey vocalizations are harmonically structured such as human vowels because the vocal mechanism in monkeys are the same as those of humans^7^,^8^,^9^,^10^,^11^. The periodic opening and closing of the vocal folds generates pulses during vocalizations. The repetition rate of the pulses determines the fundamental frequency (F0) of the vocalization and is perceived as pitch. As pulses created by the vocal folds pass through the vocal tract, the vocal tract characteristics (VTC) produce resonances and enhance/dampen particular frequency bands; these are called the formants. It has been well documented that both pitch and formant are highly important in primate communications, whereas how each acoustic characteristic contributes to vocal identification is not fully understood.

Several lines of evidence suggest that the formants created by the filter characteristics of the VTC play significant roles in the acoustic distinctiveness of individual primates, including humans. Bachorowski and Owren^12^ analysed phonemes of speech in humans and showed that vocal tract filtering may contribute to individual identification. Owren et al.^13^ analysed the vocalizations of female chacma baboons (*Papio ursinus*) and suggested that the acoustical features of vocal tract filtering may reflect individuality. The resonance of vocal tract filtering may affect individual identification in rhesus macaques^14^ and lemurs (*Eulemur rubriventer*)^15^. In addition to the formants, statistical analyses of the acoustic features of the F0, such as the beginning frequency and maximum frequency, indicate that the F0 can be a reliable cue for identifying callers in several monkey species^16^,^17^. In relatively recent research by Ceugniet and Izumi, Japanese macaques were trained to discrimination the vocalizations of different monkeys, and the subjects responded to the F0 as a discriminant stimulus for the task, which suggests that the F0 contributes to individual discrimination^18^.

In the present study, we used the contact calls of Japanese macaques to study individual vocal recognition. Green^7^ acoustically analysed and classified the vocalizations of Japanese macaques in the field and reported that Japanese macaques have several types of call. As a result of Green’s work, many other research groups have also focused on studying vocalization behaviours, and the Japanese macaque has become one of the most valuable and well-studied non-human primate models. These macaques exchange a coo call with one another when listening to the calls of other troop members^19^. The function of vocal exchange has been discussed in terms locating other individuals and maintaining within-group communication^7^. This study was performed to investigate the relative importance of acoustic cues (i.e., formant and pitch) in individual vocal recognition in Japanese macaques. We used operant conditioning and speech-processing techniques to systematically compare and quantify the perceptual contribution of each acoustic parameter.

## Results

**Figure 1:**
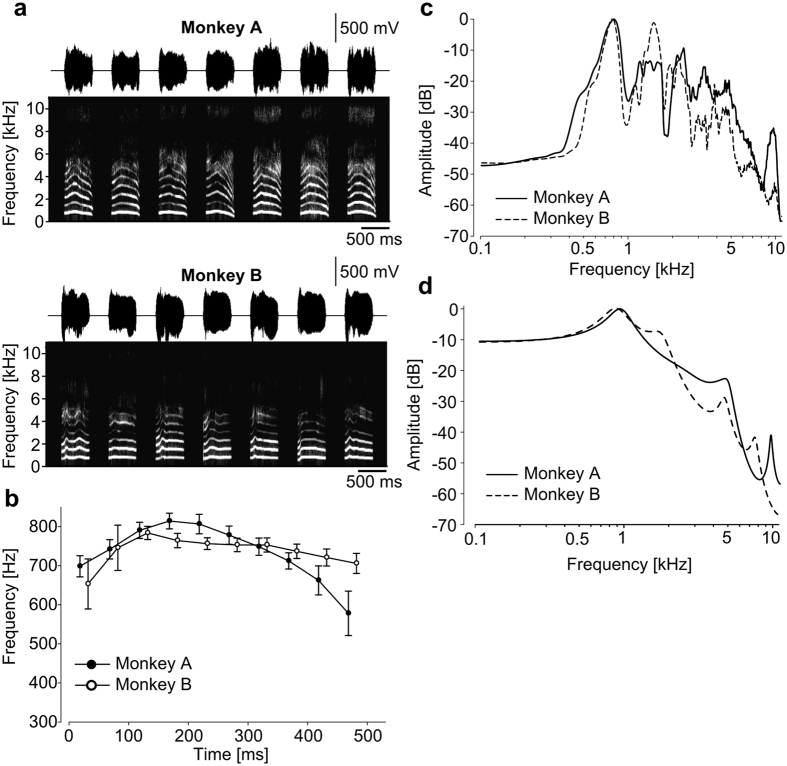
Acoustic characteristics of the stimulus coo calls. (**a**) Spectrograms of the coo calls from the two monkeys. Top panel: the coo calls of Monkey A (cooA). Bottom panel: the coo calls of Monkey B (cooB). These monkeys were unfamiliar to the subjects, and the recorded calls were modified such that they had the same durations, amplitude envelopes, and average fundamental frequencies (F0s). The subjects were trained to discriminate between the cooAs and cooBs. The right-most calls were used to synthesize the test stimuli. (**b**) Temporal pitch patterns of the coo calls of the two monkeys. Closed circles: the mean temporal pitch pattern of the coo calls of Monkey A; open circles: those of Monkey B. Error bars: standard deviations. Although the F0s were normalized, the two stimulus sets varied in terms of both the end frequency and the time of the F0 peak. (**c**) Power spectra of the cooA (solid line) and cooB (dash line) stimuli. (**d**) Linear predictive coding spectra of example cooA (solid line) and cooB (dash line). The data illustrate the differences in the vocal tract characteristics (VTCs) of the two monkeys.

**Figure 2:**
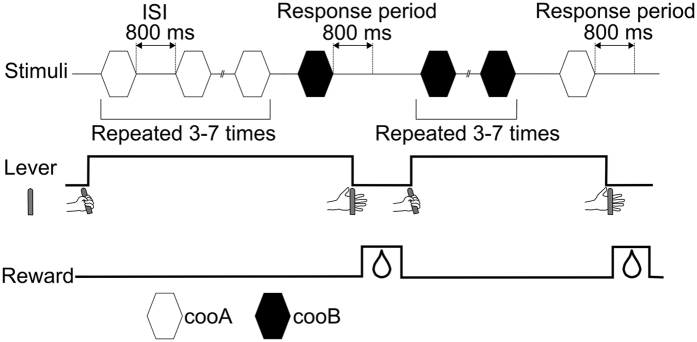
Schematized trial event sequence. Upper trace: the timing of the stimulus. Middle trace: the response of the animal. Lower trace: the timing of the reward. The subjects were required to depress a lever switch for 200 ms to begin the trial. Then, cooA (open hexagon: NoGo stimulus) was presented 3–7 times with an interstimulus interval (ISI) of 800 ms. During the repetitions, the type of cooA (out of the total of six, Fig. 1a) and the intensity of the stimulus (57, 60, and 63 dB SPL) were randomly changed. The subjects were required to continue depressing the lever while cooA was repeated. If cooB (Go stimulus) was presented, the subjects were required to release the lever within 800 ms after the offset of the cooB to receive a reward. After a correct response to a Go stimulus, the stimulus contingencies were reversed in the next trial. That is, cooA became the Go stimulus, and cooB became the NoGo stimulus. In the test trials, cooA was replaced with a test stimulus, and the stimulus was presented after cooBs were repeated as the NoGo stimuli. Neither a reward nor a punishment followed the test trial.

**Figure 3:**
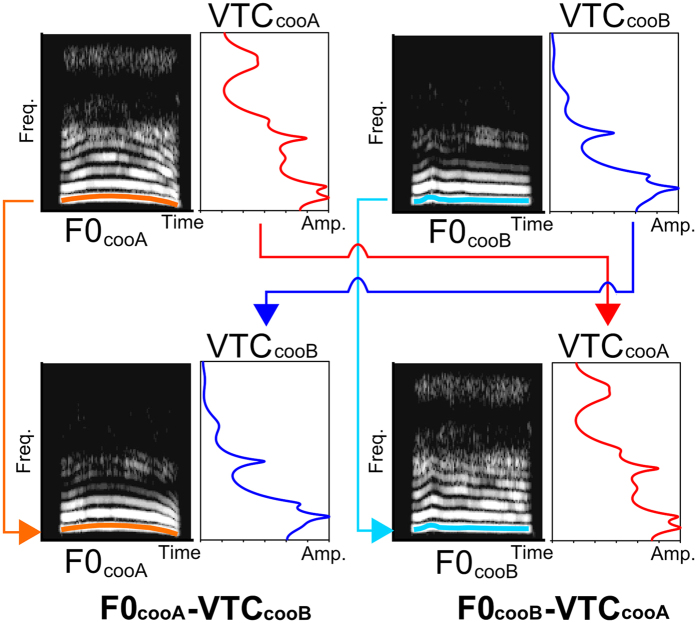
Methods for the synthesis of the stimuli. The test stimuli were synthesized by combining the F0s and the VTCs from different animals. Orange line: the F0 of Monkey A; light blue line: the F0 of Monkey B. Red line: the linear predictive coding spectrum of Monkey A; blue line: the linear predictive coding spectrum for Monkey B. F0_cooA_-VTC_cooB_ (bottom left) was synthesized from the F0 of Monkey A (orange) and the VTC of Monkey B (blue), whereas F0_cooB_-VTC_cooA_ (bottom right) was created from the F0 of Monkey B (light blue) and the VTC of Monkey A (orange).

Two Japanese macaques (subject 1 and subject 2) were trained to discriminate the coo calls of Monkey A (cooA, supplemental audio 1) and Monkey B (cooB, supplemental audio 2) with standard Go/NoGo operant conditioning (Fig. 1). Both the cooAs and cooBs were recorded from unfamiliar monkeys, meaning that the subjects had no prior experience with either cooA or cooB. The trial began when the monkey pushed a lever. The subjects were required to continue to depress the lever while the calls from the same monkey were presented repeatedly (NoGo trial). When the stimulus was changed from one monkey to another (Go trial), the subjects had to release the lever within 800 ms from the offset of the stimulus (Fig. 2) to receive a reward. The test stimuli were synthesized by combining the F0 of one individual and the vocal tract characteristics (VTC) of the other individual (Fig. 3, supplemental audio 3 and 4, F0_cooA_-VTC_cooB_ was synthesized from the F0 of cooA and the VTC of cooB, whereas F0_cooB_-VTC_cooA_ was generated from the F0 of cooB and the VTC of cooA). All of the test stimuli were presented after cooB was repeated. Both the Go response rates and reaction times (RTs) were measured to quantify the perceptions. In this procedure, a higher Go response rate and shorter RT to a test stimulus suggested that the stimulus was perceptually more similar to cooA.

**Figure 4:**
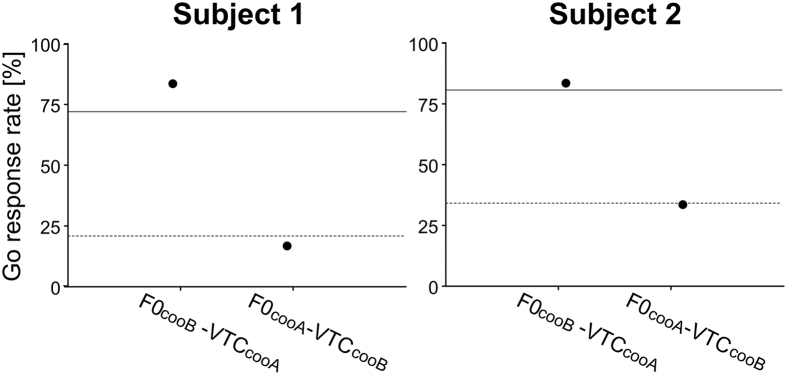
Go response rates to the test stimuli. The Go response rates to F0_cooB_-VTC_cooA_ (subject 1: 83.3%, subject 2: 83.3%) of each monkey were higher than the Go response rates to F0_cooA_-VTC_cooB_ (subject 1: 16.7%; subject 2: 33.3%). Both monkeys responded to F0_cooA_-VTC_cooB_ as they did to a coo call of Monkey B, whereas they responded to F0_cooB_-VTC_cooA_ as they did to a coo call of Monkey A. The solid line and the dotted line represent the Hit and FA rate of the test day, respectively.

Subject 1 and 2 needed 20 and 25 days of trainings respectively to learn to distinguish between the sets of cooAs and cooBs. Two days before the test day, the monkeys scored correct response rates of 82% (subject 1: d’ = 1.85, Hit = 80%, FA = 16%) and 76% (subject 2: d’ = 1.38, Hit = 75%, FA = 24%). The day before the test day, the correct response rates were 78% (subject 1: d’ = 1.54, Hit = 75%, FA = 19%) and 71% (subject 2: d’ = 1.13, Hit = 77%, FA = 65%). The Go response rates to the training stimuli in the test day did not differ from those in the training day. In the test day, the correct response rates of subject 1 and subject 2 to the training stimuli were 76% (d’ = 1.49, Hit = 72%, FA = 20%) and 73% (d’ = 1.30, Hit = 81%, FA = 34%), respectively, suggesting that the subjects maintained the same discriminatory performance with the training stimuli throughout the experiment. The Go response rates to the test stimuli for the two monkeys are illustrated in Fig. 4. The Go response rates to F0_cooA_-VTC_cooB_ (Fig. 4), which had the same F0 as the Go stimulus (=cooA) and the same VTC as the NoGo stimulus (=cooB), of subjects 1 and 2 were 16.7% and 33.3%, respectively. The Go response rates of subjects 1 and 2 to F0_cooB_-VTC_cooA_ (Fig. 4) were 83.3% and 83.3%, respectively. Our data revealed that F0_cooB_-VTC_cooA_ triggered more Go responses from both monkeys than F0_cooA_-VTC_cooB_.

**Figure 5:**
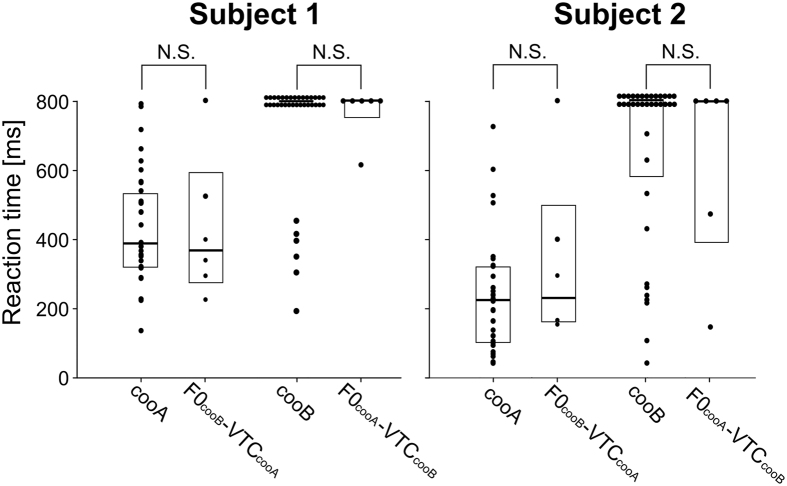
Comparisons of the reaction times (RTs) to the training and test stimuli for the two subjects. There were no significant differences in the RTs to F0_cooB_-VTC_cooA_ (subject 1: 368 (276–592) ms, subject 2: 230 (161–499) ms, median (interquartile range)) and cooA (subject 1: 416 (351–558) ms, subject 2: 226 (108–321) ms) in the training trials or in the RTs to F0_cooA_-VTC_cooB_ (subject 1: 800 (753–800) ms, subject 2: 800 (391–800) ms) and cooB (subject 1: 800 (800–800) ms, subject 2: 800 (581–800) ms) in the training trials for the two subjects. Box plots represent the median (horizontal line) and interquartile range (box) of the indicated distribution. Each plot point represents the reaction time of each trial. N.S.: not significant.

The RTs to the test stimuli were examined to quantify the perceptual similarity of the stimuli^20^,^21^,^22^,^23^. The median RTs of subjects 1 and 2 to F0_cooA_-VTC_cooB_ were 800 (interquartile range: 753–800) ms and 800 (391–800) ms, respectively. In contrast, the median RTs of subjects 1 and 2 to F0_cooB_-VTC_cooA_ were 368 (276–592) ms and 230 (161–499) ms, respectively (Fig. 5). The median RTs to F0_cooA_-VTC_cooB_ and F0_cooB_-VTC_cooA_ were compared with those to the training stimuli. Because the test stimulus was 60 dB sound pressure level (SPL), the training stimulus with same 60 dB level was treated as a comparison stimulus. The stimulus was presented 40 and 45 times to subjects 1 and 2, respectively, in the test day. Of those repetitions, 3 (in subject 1) and 4 (in subject 2) presentations were excluded from the analyses because the monkeys’ heads were not oriented towards the speaker during the presentations. The RTs to F0_cooB_-VTC_cooA_ were not significantly different from those to the Go stimulus (cooA) in either subject 1 (F0_cooB_-VTC_cooA_: 368 (276–592) ms, Go stimulus: 416 (351–558) ms; *p* = 0.93) or subject 2 (F0_cooB_-VTC_cooA_: 230 (161–499) ms, Go stimulus: 226 (108–321) ms; *p* = 0.33). Additionally, the median RT of subject 1 to the NoGo stimulus was 800 (800–800) ms and that of subject 2 was 800 (581–800) ms. There were no significant differences between the RTs of either subject to F0_cooA_-VTC_cooB_ and the NoGo stimuli in the test day (Fig. 5, subject 1:*p* = 0.93; subject 2: *p* = 0.88).

## Discussion

We used acoustic synthesis and analysis software to systematically quantify the relative importance of acoustic characteristics (i.e., the VTC and the temporal structure of the F0) when the monkeys identify callers. The behavioural data suggest that the animals perceived the F0_cooA_-VTC_cooB_ as the same as cooB, whereas they perceived F0_cooB_-VTC_cooA_ as the same as cooA instead of recognizing them as intermediate between the two stimuli. When only the VTC was switched from one type to the other, the subjects still responded as if the call type had transitioned, whereas the animals did not respond if only the temporal pattern of F0 changed (Fig. 4). The subjects’ behavioural responses revealed that the VTC played a critical role in distinguishing the stimulus sets, suggesting that monkeys relied more on the VTC than on the temporal pitch patterns in discriminating caller identity. The difference in the temporal pattern of the F0 may have been too small to enable the monkeys to differentiate the stimulus set, but we believe that this was not the case. Hopp et al.^24^ studied the sensitivity of Japanese macaques to the peak position of F0 in synthesized coo calls and demonstrated that trained animals were able to detect changes in the peak position of as little as 20–50 ms in smooth early high coos. The F0 of the cooA peak was earlier than that of the cooB peak by approximately 60 ms (the peak position of the vocalizations of Monkey A was 195 ± 22 ms and that of Monkey B was 134 ± 45 ms [average ± standard deviation]). Thus, the subjects were able to distinguish the stimulus sets using the peak position of the vocalizations in this experiment.

Monkeys are also able to discriminate vocalizations using the end frequencies of the stimuli. A previous study using pure-tone bursts of 1000 Hz revealed that Japanese macaques are able to distinguish frequency differences as small as 33 Hz (i.e., a difference of approximately 3%)^25^. In our stimulus set, the mean frequencies of the stimuli were normalized, and the temporal patterns of F0 were maintained (Fig. 1b). Therefore, the end frequencies of cooA were lower than those of cooB by approximately 120 Hz (cooA: 578 ± 57 Hz; cooB: 706 ± 26 Hz) or 15%. Thus, it is reasonable to assume that the subjects were able to distinguish the stimulus sets according to the end frequency in addition to the peak timing.

There are still several questions that remain to be answered. Whereas the past studies described above suggest that the monkeys were able to discriminate our stimulus sets by the temporal patterns of F0. It is probable that the F0 differences were sufficiently salient for use as discriminative cues compared with the VTCs. In contrast, the significance of the VTCs in the monkeys’ discrimination does not necessarily mean that the VTC is only cue that used for individual discrimination. To address these questions, we would need to quantify the contribution (if any) of the F0 to the discrimination using synthesized calls without differences in VTC (i.e., vocal signals with the same VTC that differ only in the F0) and also measure the perceptual threshold of the F0 components. In addition to those studies, because our data demonstrated that the speech-processing techniques (STRAIGHT^26^) provide reliable behavioural data, we can now create a stimulus continuum between different individuals and systematically investigate the relationships between the acoustic parameters and vocal identification.

As described in non-primate species^27^,^28^, the formants embedded in the acoustic structures of nonhuman primate calls provide cues about the physical characteristics of the caller^8^,^12^,^13^,^27^,^28^. A previous study using a preferential looking paradigm suggested that untrained rhesus monkeys use formants as indexical cues of age-related body size^29^. Fitch and Fritz^30^ also demonstrated that nonhuman primates can perceive formant shifts in species-specific vocalizations. Owren^31^,^32^ demonstrated that trained vervet monkeys can use formants to discriminate between their alarm calls in a manner similar to that used by humans to distinguish speech sounds. Similar to humans, with training, Japanese macaques exhibit exquisite sensitivity to different formant frequencies^33^. These results indicate that formants are biologically significant in the vocal communication of many primate species.

In addition to formants, pitch has also been demonstrated to be important for communication. Japanese macaques are regarded as sensitive to the temporal patterns of the F0, particularly in coo calls, because the peak temporal position differentiates the call type; i.e., smooth early high and smooth late high^34^. The F0 has also been reported to differ between individuals in several primate species, and the F0 is a statistically significant determinant of caller identity^16^,^17^. To our knowledge, however, there have been only a few attempts to directly compare the importance of the VTC and F0 in identification. Ceugniet and Izumi^18^ trained two Japanese macaques to discriminate the vocalizations of different individuals using operant conditioning; these authors demonstrated that macaques judge individuality via a combination of both the VTC and the frequency of the F0. Thus, the dominant acoustic cues in the determination of individuality in non-human primates are still largely unknown. Our data indicated that the formant frequencies generated by the VTC were preferentially used over the F0 temporal structures to discriminate the stimulus sets, which strengthens the suggestion that the formant structure is significant for the perception of conspecific sounds and also possibly for individual identification.

This experiment was performed to determine the primary cues that are used for the identification of individuals. However, the monkeys may have only *discriminated* between the features of two sets of vocalizations rather than *identifying* the individual the caller. Further studies are required to determine whether monkeys perceive the stimulus sets as the vocalizations of two different monkeys.

## Conclusions

Many primates, including humans, can discriminate individuality based only on listening to vocalizations. Our experiments directly compared the relative importance of acoustic parameters in Japanese macaques, and the results suggest that VTCs are more important for discriminating the caller than the temporal structure of the fundamental frequency.

## Materials and Methods

### Subjects

Two male Japanese macaques (*Macaca fuscata*) were used in this experiment. At the time of testing, subject 1 was 7 years old and subject 2 was 10 years old. Each animal was kept in an individual primate cage under a constant 13-h/11-h light/dark cycle. Their access to liquids was limited because water served as the positive reinforcement in the experiments. All procedures were conducted in accordance with guidelines established by the Ethics Review Committee of Doshisha University, and the experimental protocols were approved by the Animal Experimental Committee of Doshisha University.

### Experimental apparatus

The training and tests were conducted in a sound-attenuated room (length × width × height of 1.70 m × 1.85 m × 2.65 m). The monkey chair in which the subjects were seated during the experiment was equipped with a drinking tube and a response lever. A loudspeaker (SX-WD1KT; Victor, Tokyo, Japan) was positioned 58 cm in front of the subject’s head at the same height as the ears. All acoustic stimuli were amplified (SRP-P2400; Sony, Tokyo, Japan), and the frequency response of the speaker was flattened (±3 dB) between 0.4 kHz and 16 kHz with a graphic equalizer (GQ2015A; Yamaha, Hamamatsu, Japan). A white light-emitting diode (LED) and a charge-coupled device (CCD) video camera were attached to the top of the speaker. An LED was lit during training and test trials to provide lighting, and subjects were monitored using the CCD camera.

### Acoustic stimuli

The sound stimuli were obtained from two adult male monkeys (Monkey A and Monkey B). The coo calls of Monkey A (cooA) and Monkey B (cooB) were recorded using a condenser microphone (type 2142; Aco, Tokyo, Japan) and digital audio tape recorder (TCD-D8; Sony, Tokyo, Japan) with a resolution of 16 bits and a sampling rate of 44.1 kHz. The monkeys (Monkey A and Monkey B) who provided the coo calls had never encountered the subject monkeys (subjects 1 and 2), and this experiment was the first time that the subjects heard the voices of the stimulus monkeys. Fourteen coo calls (seven from each monkey) with signal-to-noise ratios > 40 dB were randomly selected from the recorded sounds.

The coo calls were analysed using STRAIGHT^26^ to measure three acoustic parameters of the coo calls: the fundamental frequencies (F0s), vocal tract characteristics (VTCs), and durations. Twelve coo calls (six coo calls per individual) of the total of fourteen were used as training stimuli (cooAs and cooBs, Fig. 1). One coo call from each monkey was not played during training, and these calls were used to synthesize the test stimuli. The test stimuli coo calls were synthesized by combining the F0s and VTCs of the different individuals using STRAIGHT. Two types of test stimulus were synthesized as probes. The F0_cooA_-VTC_cooB_ stimulus was synthesized from the F0 of cooA and the VTC of cooB, whereas the other test stimulus, F0_cooB_-VTC_cooA_, was generated from the F0 of cooB and the VTC of cooA (Fig. 3). The call durations were equalized to 517 ms (i.e., the average of all of the calls) via linearly time-stretching or compressing with STRAIGHT. With this manipulation, the duration of the original call was modified by 10% in the most extreme case. The root-mean-square (RMS) envelopes were calculated with a 512-point (≈12 ms) window, and the amplitude envelopes of all calls were normalized to average shape (Fig. 1a). The overall amplitudes of stimuli were digitally modified and calibrated (with a microphone: type 7016; Aco) at to yield three different sound pressure levels (SPL, *re*: 20 μPa), i.e., 57, 60, and 63 dB, at the position of the head. That is, three different SPL stimuli were generated for each stimulus type. The fundamental frequencies of all of the calls were also modified, and the temporal average of the F0 was normalized to 733 Hz (i.e., the average of all of the original calls, Fig. 1b), and the vocal tract characteristics remained unmodified (Fig. 1c,d). In this study, we only use the synthesized stimulus for a test. Untrained cooA and B were never presented to the subjects, and were saved for a subsequent report.

### Procedure

We employed standard Go/NoGo operant conditioning in this study. The event sequence of the trials is schematically illustrated in Fig. 2. The subjects were required to depress the lever switch on the monkey chair for 200 ms to begin the trial. Then, the calls from a single subject, either Monkey A or Monkey B, were repeated 3–7 times. In each repetition, the call type was randomly selected from 18 different types of call (6 types of coo call × 3 intensities from the same monkey). The interstimulus interval between adjacent stimuli was 800 ms. While the calls from the same monkey were presented (NoGo trial), the subjects were required to continue depressing the lever (correct rejection: CR). In other words, after a CR response, the next stimulus automatically began as long as an animal continued to hold the lever. After 3 to 7 repetitions, the stimulus was changed from one monkey to the other (Go trial). The subjects were required to release the lever within 800 ms of the offset of the stimulus (Hit). After a Hit response, the next trial did not begin until an animal depressed the lever again.

For example, a trial began with the repetitive playback of cooAs (NoGo stimulus). In the repetition, the individual cooA (of the total of six) and the intensity of the stimulus (57, 60, and 63 dB SPL) were changed randomly. The subjects were required to continue depressing the lever while cooA was repeated. When cooB (Go stimulus) was presented, the subjects were required to release the lever within 800 ms after the offset of the cooB. Hits were reinforced with 2 ml of fruit juice. When the subjects released the lever during the repetition period of the NoGo stimulus (false alarm: FA) or failed to release the lever within 800 ms after the Go stimulus (miss), a 15–20 s timeout period accompanied by the turning off of the LED was provided as feedback. After an FA or miss response, a trial with same stimulus contingencies was provided. When the timeout period was over, the LED was lit to inform the animal of the initiation of a new trial. If the subject responded successfully to the Go stimulus, the stimulus contingencies were reversed in the next trial. That is, the next trial began with the playback of cooB instead of cooA, and the subject had to release the lever when cooA was played to receive the reward. Performance was measured as the correct response percentage (CRP: the total percentage of the Hits and CRs). One hundred thirty to 160 Go trials (i.e., trials in which the stimulus changed from one monkey to the other) and 650 to 800 NoGo trials were presented per day to both subjects. After the subjects’ scores exceeded the CRP threshold (70%) for two consecutive days, they proceeded to the test day. A test stimulus was presented, after cooB was repeated 5 times, and each type of test stimulus was played 6 times. The test trials were interleaved with 10–20 training trials. Neither reward nor punishment followed the test trial.

### Statistical analysis

We measured both the Go response rates and RTs (the time period between the end of each stimulus and the release of the lever switch). If the subjects did not release the lever within the 800 ms response period, the RT was regarded as 800 ms for the analysis. The CCD camera on the speaker allowed us to monitor the behaviour of each subject, and if the subject did not look straight into the speaker during the sound playback, the data in the trial were excluded from the analysis. The RTs to the test (F0_cooB_-VTC_cooA_ and F0_cooA_-VTC_cooB_) and training stimuli were analysed by Mann-Whitney U test using a commercial statistical software package (SPSS 21; IBM Armonk, NY, US).

## Additional Information

**How to cite this article**: Takafumi, F. *et al*. Role of vocal tract characteristics in individual discrimination by Japanese macaques (*Macaca fuscata*). *Sci. Rep.* **6**, 32042; doi: 10.1038/srep32042 (2016).

## Supplementary Material

Supplementary Information

Supplemental Audio 1

Supplemental Audio 2

Supplemental Audio 3

Supplemental Audio 4
