# Supplementary material for: Role of vocal tract characteristics in individual discrimination by Japanese macaques (Macaca fuscata)
Source: Sci Rep. 2016 Aug 23;6:32042. doi: 10.1038/srep32042 (PMC4994087; doi:10.1038/srep32042)
Supplement: Supplementary Information [file srep32042-s1.doc]

**Supplemental Information**

Title: **Role of vocal tract characteristics in individual discrimination by Japanese macaques (*Macaca fuscata*)**

Authors: Takafumi Furuyama*, Kohta I Kobayasi, Hiroshi Riquimaroux*

Primary affiliation (of all authors): Doshisha University

Postal address: 1-3 Miyakotani Tatara, Kyotanabe, Kyoto, Japan, zip 610-0321

E-mail address:

takafumifuruyama@gmail.com (TF),

hrikimar@mail.doshisha.ac.jp, hiroshi_riquimaroux@brown.edu (HR)

**Supplemental Audio 1:** (File name: Sounds of cooA.wav). Coo calls of Monkey A (cooAs) used in the experiment.

**Supplemental Audio 2:** (File name: Sounds of cooB.wav). Coo calls of Monkey B (cooBs) used in the experiment.

**Supplemental Audio 3:** (File name: Test stimulus (F0cooA-VTCcooB).wav). Test stimulus (F0_cooA_-VTC_cooB_) synthesized from the F0 of cooA and the VTC of cooB.

**Supplemental Audio 4:** (File name: Test stimulus (F0cooB-VTCcooA).wav). Test stimulus (F0_cooB_-VTC_cooA_) generated from the F0 of cooB and the VTC of cooA.
